# Putting comprehensive genomic profiling of ctDNA to work: 10 proposed use cases

**DOI:** 10.1016/j.jlb.2024.100140

**Published:** 2024-01-17

**Authors:** Aakash Desai, Lincoln W. Pasquina, Candice Nulsen, Rachel B. Keller-Evans, Douglas A. Mata, Hanna Tukachinsky, Geoffrey R. Oxnard

**Affiliations:** a Division of Hematology and Oncology, Department of Medicine, University of Alabama at Birmingham, AL, USA; b Foundation Medicine, Inc., Boston, MA, USA; c Boston Medical Center, Boston, MA, USA

**Keywords:** Circulating tumor DNA, Comprehensive genomic profiling, Liquid biopsy

## Abstract

Liquid biopsy profiling of circulating tumor DNA (ctDNA) has become established as a compelling, pragmatic diagnostic in the care of cancer patients and is now endorsed by multiple cancer care guidelines. Moreover, ctDNA profiling technologies have advanced significantly and offer increasingly comprehensive and reliable insights into cancer. In this review, we focus on applications of ctDNA and propose that a critical untapped opportunity is in considering how we utilize these accessible, scalable technologies across diverse potential applications. With a specific focus on clinical applications, rather than research uses, we describe 10 use cases for ctDNA profiling across four categories: (1) established and (2) emerging applications of ctDNA profiling for therapy selection, (3) incidental detection of secondary genomic findings, and (4) quantification of plasma DNA tumor content.

## 1 Introduction

**Fig. 1:**
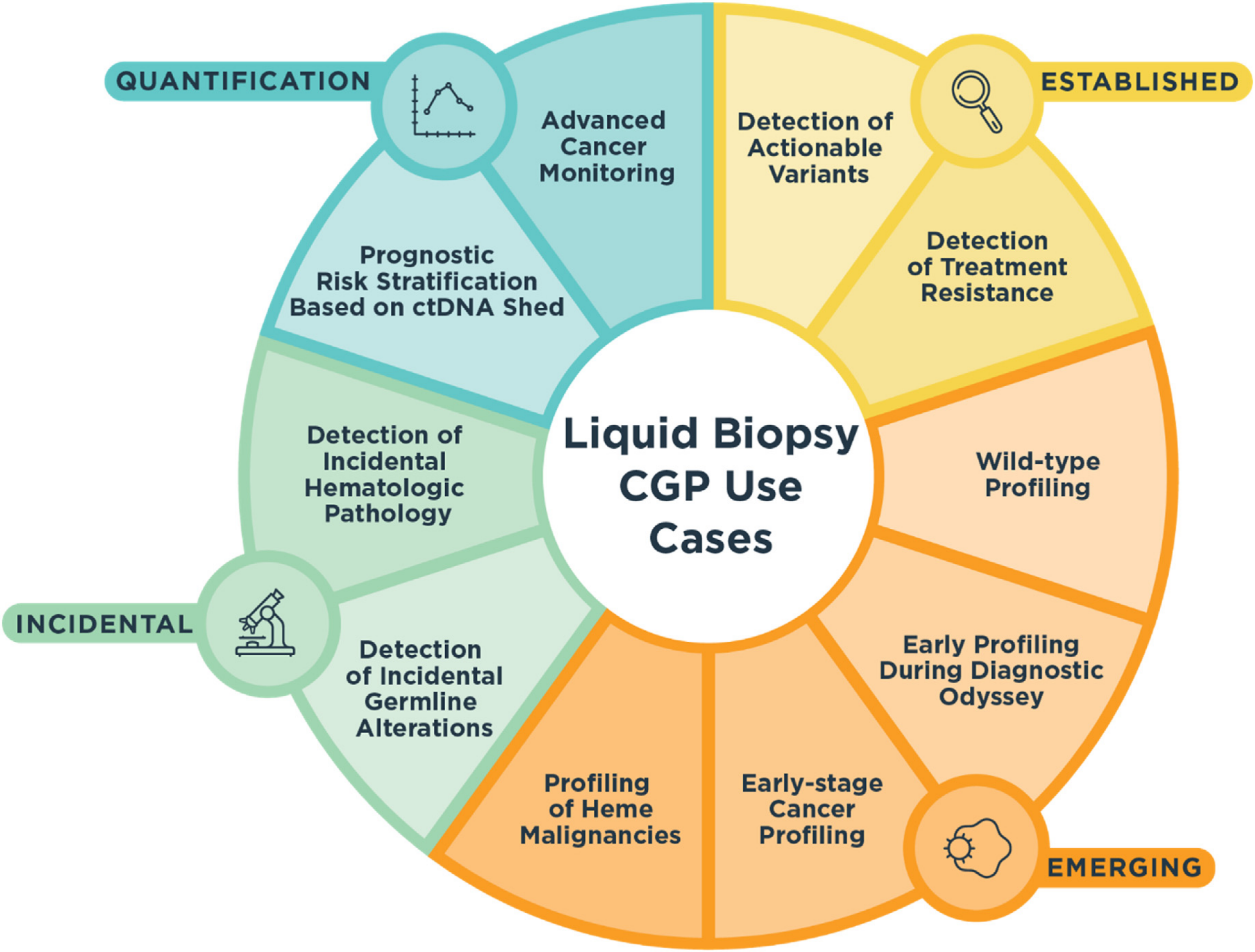
**Liquid Biopsy CGP Use Cases.** Broadly, use cases fall into the categories of Established, Emerging, Incidental, and Quantification. Established use cases include detection of actionable variants and detection of treatment resistance. Emerging use cases include wild-type profiling, early profiling during the diagnostic odyssey, early-stage cancer profiling, and profiling of heme malignancies. Incidental use cases include detection of incidental germline alterations and detection of incidental hematologic pathology. Quantification use cases include prognostic risk stratification based on ctDNA shed and advanced cancer monitoring. CGP, comprehensive genomic profiling; ctDNA, circulating tumor DNA.

The accessibility of tumor deoxyribonucleic acid (DNA) shed into the blood makes it a compelling analyte for informing multiple aspects of a patient's cancer care [1,2]. Evaluating a patient's blood for circulating tumor DNA (ctDNA), known as liquid biopsy, enables the assessment of disease states along multiple axes, including diagnosis, prognosis, treatment selection, and monitoring [[3], [4], [5], [6]]. In this review, we focus on comprehensive genomic profiling (CGP) via liquid biopsy, which we define as any next-generation sequencing (NGS) assay that profiles multiple genes from cell-free DNA (cfDNA) for the purpose of characterizing cancer. Importantly, CGP is distinct from blood-based assays that determine the presence or absence of cancer, either for molecular residual disease (MRD) detection or cancer screening, both of which are outside the scope of this review. Liquid biopsy-based CGP has well-established and emerging uses to address a range of clinical decision points in oncology. We review the role of ctDNA CGP in this growing range of use cases, including therapy selection, risk stratification, and disease state monitoring (Fig. 1). With the intent of advancing clinical utility and investigation, we focus on near-term clinical use cases and avoid discussion of research-only applications of liquid biopsy, such as ctDNA-based whole exome/genome sequencing. We acknowledge that there is a range of liquid biopsy CGP assays available for clinical use, each with technical attributes with which users should be familiar. Indeed, each broadly available assay covers a different number of genes [[7], [8], [9], [10], [11]] and each to a varying degree, resulting in differing abilities to detect rearrangements [12] and genome-wide signatures such as tumor mutational burden (TMB) [[13], [14], [15]], reviewed more fully below. In this review, we summarize evidence supporting 10 distinct use cases in which ctDNA CGP may have near-term clinical utility, capture some of the technical differences between clinically available ctDNA CGP assays, and discuss challenges and future directions for CGP in liquid biopsy.

## 2 Established uses of ctDNA profiling for therapy selection

### 2.1 Detection of actionable variants

CGP plays an established role in treatment selection for advanced cancers with multiple potential targeted agents [16]. Indeed, CGP of solid tumors for treatment selection has been endorsed by societal guidelines in the care of multiple disease types [[17], [18], [19], [20]]. CGP of liquid biopsies represents a pragmatic alternative to tissue testing, particularly in situations where tissue is unavailable, when biopsy would cause significant morbidity, or when biopsy is unlikely to yield a viable specimen [21]. In non-small cell lung cancer (NSCLC), where several molecularly targeted therapy options are available and tissue is challenging to obtain in sufficient quantities, CGP via liquid biopsy is increasingly relevant and is often considered complementary to tissue testing [22]. While early liquid biopsy approaches focused on single genes such as epidermal growth factor receptor (*EGFR*) [23], these have rapidly been eclipsed by multigene assays, given the growing number of actionable biomarkers both at lung cancer diagnosis and for treatment resistance.

A critical feature of liquid biopsy is its high specificity, even when detecting actionable variants at a low variant allele frequency (VAF) [23], thereby enabling actionable positive findings with a high positive predictive value (PPV) [24]. The high PPV is powerful when combined with the reliable turnaround time, which is significantly faster for CGP of liquid biopsy compared with that of tissue [25]. This can allow for faster treatment initiation and avoid unnecessary exposure to ineffective therapies [26,27].

The primary limitation of CGP of liquid biopsies is that the overall sensitivity for driver alterations has consistently been lower compared with that of tissue [28]. A review of the United States (US) Food and Drug Administration (FDA) labels for approved liquid CGP assays shows that the positive percent agreement compared with tissue testing ranges between 55% and 100%, depending on the sample count and alteration type for each individual alteration-level analysis [[29], [30], [31], [32], [33]]. The sensitivity of liquid biopsy is a function of ctDNA shed into the blood, which can vary significantly across cancer types and disease states [4]. Given this variable sensitivity, negative liquid biopsy results should be interpreted cautiously and should ideally be confirmed with tissue testing [6].

### 2.2 Detection of resistance alterations

Understanding the mechanisms of resistance to targeted agents is vital for personalizing therapies to overcome drug resistance. As resistance is inherently an acquired disease state requiring a new sample for analysis, liquid biopsy CGP represents an intuitive opportunity to assess these resistance mechanisms. The variable sensitivity of liquid biopsy described previously in *2.1. Detection of actionable variants* continues to apply to drug resistance, meaning that a negative liquid biopsy may require a follow-on tissue biopsy to assess for missed resistance alterations [34]. Interestingly, owing to the heterogeneity of resistance, it is acknowledged that resistance alterations may be detected via liquid biopsy only and may not be present in a paired tissue biopsy result [35]. A growing number of resistance alterations detected in ctDNA can be targeted therapeutically. This paradigm began with the use of osimertinib to overcome the *EGFR* exon 20 p.T790M mutation in *EGFR*-mutant NSCLC, where outcomes were similarly favorable in patients with p.T790M detected in tissue or liquid biopsy [34]. Similarly, newer *EGFR* resistance alterations such as exon 20 p.C797S can be overcome therapeutically [36]. In anaplastic lymphoma kinase (ALK) fusion-positive NSCLC, detection of a secondary resistance mutation such as exon 23 p.G1202R was associated with increased responsiveness to lorlatinib [37]. This paradigm now applies to breast cancer as well, with detection of an estrogen receptor 1 (*ESR1*) mutation in liquid biopsy being associated with a benefit from elacestrant therapy [38]. Additionally, other resistance alterations detected in liquid biopsy may indicate therapies that should be avoided, such as acquired rat sarcoma (*RAS*) mutations in colorectal cancer [39] or breast cancer gene (*BRCA*) reversion mutations in patients with a homologous recombination deficiency (*HRD*) mutation [40,41]. Detection of multiple co-existent resistance mechanisms in liquid CGP is an increasingly recognized phenomenon, such as has been described following resistance to Kirsten rat sarcoma (KRAS) exon 2 p.G12C inhibitors [42], and is acknowledged as a challenging diagnostic finding that precision therapies may be unable to overcome. Notably, tissue testing may still be indicated specifically in cases where transformation of cancer to a different histology is a concern, for example, small cell transformation of *EGFR*-mutated NSCLC after treatment with tyrosine kinase inhibitors [43] or prostate adenocarcinoma into small cell neuroendocrine cancer under androgen deprivation therapy [44].

## 3 Emerging uses of ctDNA profiling for therapy selection

### 3.1 Wild-type profiling

As described in “2. Detection of resistance alterations,” it is difficult to have confidence in a “negative” liquid biopsy result; however, having confidence in negative results may be necessary to confirm wild-type status when selecting therapies and for determining eligibility of patients in various clinical trials. For example, immune checkpoint inhibitors (ICIs) can be very effective in advanced NSCLC (aNSCLC), but are only indicated in *EGFR*/*ALK*-negative cancers [45]. Clinical trials of ICIs in early-stage and advanced NSCLC often require confirmation of the absence of *EGFR* and *ALK* alterations, which liquid biopsy typically cannot confirm. Similarly, *anti*-EGFR antibody therapy in advanced colorectal cancer is indicated only in the absence of a *RAS* alteration [46].

Technological solutions are needed and are in development to enable confirmation of wild-type status by using liquid biopsy. Given that liquid biopsy has variable sensitivity, reporting a confident negative result requires identification of circumstances where high sensitivity can be established. For example, in a pilot analysis, Meador *et al.* showed that the sensitivity for *EGFR* exon 20 p.T790M alterations was overall modest (∼80%) in liquid biopsy, but exceeded 90 % when limited to cases where an *EGFR* driver alteration was detected at >1% VAF [47]. When sensitivity is high, a negative result can be more confidently reported, potentially avoiding the need for a confirmatory biopsy.

Broadening this approach beyond *EGFR*-mutant lung cancer requires robust methods for estimating ctDNA content, such as the calculation of ctDNA tumor fraction (TF), an algorithmic method that can provide information on ctDNA content even without the detection of pathogenic variants [4,7]. When ctDNA TF is elevated, the sensitivity for driver alterations is increased such that a driver-negative tumor can be distinguished from a sample that merely has low amounts of ctDNA, reducing the need for confirmatory tissue testing in some situations [48].

### 3.2 Early profiling during diagnostic odyssey

Liquid biopsy CGP is uniquely different from tissue CGP in that it can be ordered based on a clinical diagnosis of advanced cancer, in parallel with determining the pathological diagnosis using tissue biopsy. Thus, CGP by liquid biopsy has been investigated during the diagnostic odyssey of cancer patients, in parallel with tissue biopsy.

In a provocative early report, Thompson *et al.* ordered a liquid biopsy at the time of diagnostic testing in 110 patients with newly diagnosed metastatic NSCLC and found that results were routinely available before the first oncology visit, shortening the time to treatment initiation [49]. Similarly, Cheng *et al.* studied liquid CGP in 20 hospitalized patients with suspected metastatic NSCLC and found that early liquid CGP reduced the time to results compared to standard workflows [50]. A more recent non-randomized study from Toronto enrolled 150 patients with radiological evidence of advanced lung cancer and offered liquid CGP; among 90 patients with advanced non-squamous NSCLC, 23% were able to start therapy before tissue NGS results were available [51].

Additionally, studies suggest that CGP could be incorporated as a diagnostic tool when tumor origin is uncertain [13,50,52]. Recent studies have proposed the incorporation of genomics into the diagnosis of certain hematological malignancies [53,54]. The presence of a transmembrane protease serine 2:v-ets erythroblastosis virus E26 oncogene homolog (*TMPRSS2-ERG*) fusion is diagnostic of prostate carcinoma [55], and many fusions are diagnostic of various sarcomas [56,57]. Certain genomic variants, individually or in combination, are less definitive but can be informative in suggesting one diagnosis over another [58]. Indeed, in a prospective trial in China, patients with clinically diagnosed lung cancer who were unable to provide tissue for pathological diagnosis received icotinib based on the presence of a plasma *EGFR* mutation (without a pathological diagnosis) with a median progression-free survival (PFS) of 10.3 months [59]. However, at present, while liquid biopsy can supplement expedited diagnosis, it cannot replace a tissue-based surgical pathology diagnosis.

### 3.3 Early-stage cancer profiling

The growth of neoadjuvant therapy paradigms across a number of cancers makes liquid biopsy CGP potentially appealing to guide precision therapy for pre-metastatic cancers. However, the utility is not well characterized in this setting, and detection rates may be lower owing to overall less shedding of ctDNA [60]. The expectation is thus that the assay sensitivity would be reduced further from the variable sensitivity of liquid biopsy already observed in advanced cancer, further increasing the risk of false negative results and the importance of complementary tissue testing. Interestingly, we expect that those early-stage patients with alterations detectable by liquid biopsy CGP may have a worse prognosis, as has been observed in the post-surgical MRD setting [61,62]. In this setting, there is an opportunity to personalize therapy and duration of treatment based on ctDNA-guided risk stratification [63]. Studies characterizing the potential value of liquid biopsy CGP in early-stage cancers are ongoing, such as the Lung Cancer Research Foundation (LCRF) LCMC4 Evaluation of Actionable Derivers in EaRly Stage Lung Cancer (LEADER) Neoadjuvant Screening Trial in stage I-III NSCLC (ClinicalTrials.gov: NCT04712877) [64] that includes both tissue and liquid CGP to determine precision neoadjuvant regimens. Additional studies are needed to further elucidate the prevalence and quantification of ctDNA shed in early cancers, as well as to characterize the clinical benefit of targeted agents in the curative setting.

### 3.4 Profiling of heme malignancies

Liquid biopsy is emerging as a potential route for assaying ctDNA attributable to a broad range of hematological malignancies and may serve as an alternative or complementary tool relative to conventional buffy coat, bone marrow aspirate, and tissue sequencing in the future [65]. Similar to solid tumors, neoplastic lymphoid cells, plasma cells, and myeloid cells shed ctDNA into the blood stream, which is detectable by liquid biopsy-based ctDNA sequencing. There is emerging interest in this approach, which may have particular utility in patients for whom tissue biopsies routinely fail by conventional tissue-based sequencing owing to low tumor nuclei cellularity. Tan *et al*. recently reviewed the clinical value of this approach, meta-analytically synthesizing data from 5 studies to demonstrate a sensitivity of 51% and a specificity of 96% for liquid biopsy as a diagnostic marker for hematological malignancies [66]. In a recent study of 271 cases of a broad range of hematological malignancies by Mata *et al.*, ctDNA liquid biopsy compared favorably with conventional buffy coat or tissue biopsy, with a positive percent agreement of liquid biopsy with paired tissue samples (73.4% vs. 73.8%) [67]. In all, 52.4% of the case pairs had genomic variants detected in the ctDNA sample only (and not on tissue analysis), underscoring the deep analytical sensitivity of liquid biopsy for detecting low-level disease clones, including clinically relevant resistance alterations such as Bruton tyrosine kinase (*BTK*) exon 15 p.C481X ibrutinib resistance mutations in chronic lymphocytic lymphoma (CLL) [68]. There is emerging interest in leveraging liquid CGP for hematological malignancies in drug development for biomarker-based trials, in part because it is practical (requiring only a peripheral blood draw) and pragmatic (capable of being performed on banked frozen plasma samples).

## 4 Incidental detection of secondary genomic findings

### 4.1 Detection of germline alterations as secondary findings

As with tissue-based CGP, ctDNA CGP may uncover pathogenic germline variants in cancer susceptibility genes (CSGs) [69,70]. These findings have significant implications for clinical management of patients (*e.g.*, targeted therapy with poly (adenosine diphosphate (ADP)-ribose) polymerase [PARP] inhibitors in germline *BRCA*-mutated breast, ovarian, prostate, and pancreatic cancer [[71], [72], [73], [74], [75]]) and their families (*e.g.*, cancer screening and risk-reducing interventions) [76,77]. In recognition of this, emphasis on the importance of reporting and confirming suspected germline variants identified through tumor profiling is reflected in several national and societal guidelines [[78], [79], [80], [81]]. Germline alterations are often readily apparent in ctDNA CGP based on the VAF. Heterozygous germline variants are commonly detected in both ctDNA and cfDNA in the range of 50% VAF, whereas somatic alterations typically occur at VAFs 1–2 orders of magnitude lower [82,83]. However, high tumor DNA levels (including high ctDNA shedding, *i.e.*, high TF) or allelic imbalance due to loss of heterozygosity or copy number variation can cause somatic variants to occur in similar VAF ranges as germline variants, such that definitive characterization of a variant as somatic versus germline is not possible using VAF alone [[82], [83], [84]].

Additional considerations when gauging the potential that a variant detected by ctDNA CGP is of germline origin include the specific CSG (*e.g.*, the probability that tumor CGP-detected variants are of germline origin is high for some genes, such as *BRCA1/2*, and low for others, such as tumor protein P53 [*TP53*] [85]) and the specific variant (*e.g.*, pathogenic founder mutations known to be associated with specific populations [84]). A strategy that takes multiple such considerations into account allows for the identification of potential germline variants with improved confidence. For example, Tung *et al*. applied a series of filters to CGP results from >125,000 tissue and liquid biopsies, identifying potentially pathogenic germline variants in 9.7% of biopsies, including 6.8% of liquid biopsies, across cancer types and in both expected (*e.g.*, *BRCA1/*2 in breast cancer) and unexpected (*e.g.*, *mutY* DNA glycosylase [*MUTYH*] outside of colorectal cancer) gene/cancer-type contexts [86]. Alternatively, computational algorithms for identifying germline versus somatic variants based on the VAF [82], modeling of local germline allele counts based on single nucleotide polymorphisms (SNPs) [83], or still more complex methods accounting for multiple factors [87] have been developed.

Importantly, while these approaches improve confidence in distinguishing potential pathogenic germline variants from ctDNA CGP, it is necessary that suspected germline variants are confirmed using dedicated germline testing with the opportunity for genetic counseling to best assess associated hereditary risk [[88], [89], [90]]. ctDNA CGP can thus serve a complementary role to clinical germline evaluation in the clinically and/or genomically informed pursuit of germline genetic testing [86].

### 4.2 Detection of incidental hematologic pathology

DNA derived from immune cells undergoing clonal hematopoiesis (CH) is another non-tumor source of detectable somatic mutations in cfDNA [91]. The most commonly mutated genes in CH are DNA methyltransferase 3 alpha (*DNMT3A*), Tet methylcytosine dioxygenase 2 (*TET2*), ASXL transcriptional regulator 1 (*ASXL1*), Protein phosphatase, magnesium-dependent, 1, delta isoform (*PPM1D*), Janus kinase 2 (*JAK2*), and splicing factor 3b Subunit 1 (*SF3B1*); however, CH alterations may also include variants in *TP53*, Ataxia-telangiectasia mutated serine/threonine kinase (*ATM*), and other genes that are both frequently CH- and tumor-derived, and their source may be difficult to distinguish. Sequencing peripheral blood mononuclear cells (PBMCs) is a methodology for identifying CH-derived genomic alterations, enabling their removal from the tumor profile [92]. However, this blood cell sequencing must be performed to a depth similar to or deeper than that of plasma profiling, and may not be able to fully account for all CH seen in the ctDNA. Tissue testing can also identify tumor-derived signals, providing further evidence for distinguishing which ctDNA alterations may or may not be CH-derived.

There is growing evidence of the potential utility of this CH signal. Although the majority of patients with CH signals in their cfDNA will not develop myelodysplastic syndromes (MDS) or myeloproliferative neoplasms, particular variants in particular genes may constitute a higher risk of undiagnosed or impending hematological disease [[93], [94], [95], [96]]. A recent study from Institut Gustave Roussy, France, reported that in 9 of 18 patients who received a hematology consultation as part of a molecular tumor board recommendation, a hematological malignancy was confirmed [97]. A case study documented the detection of high-risk CH variants *DNMT3A* exon 23 p.R882H and isocitrate dehydrogenase 2 (*IDH2*) exon 4 p.R140Q at a low VAF in a patient with prostate cancer who developed acute myeloid leukemia a year later, driven by the same mutations [98].

Detection of CH variants might be used to inform treatment and follow-up surveillance decisions. Presence of particular types of CH (mutations in DNA repair genes and *TP53*) may predispose patients to the development of therapy-related myeloid neoplasms [99], predict outcomes after autologous stem cell transplantation [100], and provide insights into the tumor microenvironment and response to immunotherapy [101,102]. Presence of CH variants in a liquid biopsy can also inform of risk for non-oncologic conditions, such as an increased risk of coronary heart disease [103] and cerebrovascular events [104].

## 5 Quantification of plasma DNA tumor content

### 5.1 Prognostic risk stratification based on ctDNA shed

Broad profiling of ctDNA has potential clinical utility beyond the identification of pathogenic alterations. Multiple studies have shown that actual levels of ctDNA in liquid biopsies have an independent prognostic value [105] and may offer an objective measure of tumor burden (*i.e.*, total tumor volume and growth rate of those tumor cells). For some diseases such as lung cancer with canonical driver alterations, detection of individual driver variants (*e.g.*, *EGFR*, *KRAS*) can offer a measure of ctDNA shed [106,107], yet the same could be feasible in other tumor types such as pancreatic [108], prostate [109], bladder [110], breast [111], and colorectal [112] cancer. The association between ctDNA shed and prognosis can lead to counter-intuitive findings; for example, in a ctDNA analysis of the ALEX trial in advanced *ALK*-positive NSCLC, patients had better outcomes if they were *ALK*-negative by ctDNA in both the alectinib and crizotinib arms [113]; such improved outcomes in “biomarker negative” patients is merely because the tissue biomarker is positive, while the ctDNA shed is low. Stratifying patients by ctDNA levels, therefore, distinguishes patients by expectation of overall survival [105], and this prognostication was found to be persistent across a wide range of cutoff values [105]. ctDNA levels could therefore be useful in stratifying patient risk, either on its own or as modifications of care nomograms [114,115].

Various methods of quantification are available, and, at the most basic level, an indication of the presence or absence of ctDNA can be useful [116]. Among methods based on normalization of ctDNA to the total concentration of cfDNA, VAF and TF are widely known. VAFs of driver alterations can be analyzed directly. Methods that consider the mean, median, or maximum somatic allele frequency (all variously referred to as MSAF), incorporating many or all detected alterations, can be more representative of the overall tumor burden than individual VAFs [117]. TF, described above, incorporates non-variant signals in addition to VAF to arrive at a quantification. Additionally, absolute measurements of ctDNA content have variously been expressed as mean tumor molecules per milliliter of blood (mtm/mL) [118], haploid genome equivalents per milliliter [119], and nanograms of ctDNA per milliliter [120].

How can ctDNA-based risk stratification be applied in clinical practice is a question that is being investigated in the ongoing randomized PROstate Cancer Treatment Optimization Via Analysis of Circulating Tumor DNA (PROTRACT) study in metastatic castrate-resistant prostate cancer (ClinicalTrials.gov: NCT04015622) [121]. In patients with progression on abiraterone treatment, 2 competing standards of care are available: docetaxel chemotherapy, with a demonstrated survival benefit but well-recognized toxicities, or enzalutamide, a second novel hormonal therapy. In this trial, patients are randomized to two approaches: in one arm, the clinician choses between these two therapies, while in the other arm patients are allocated by ctDNA levels, with higher TF receiving chemotherapy and lower TF receiving hormonal therapy. The goal is to prolong PFS by ensuring that higher-risk patients with high TF receive docetaxel chemotherapy [121]. Such a paradigm of treatment intensification for cases with higher tumor burden may resonate in a number of areas of oncology, especially with the growing use of high potency antibody drug conjugates.

### 5.2 Advanced cancer monitoring

Levels of ctDNA may have additional utility in longitudinal monitoring of treatment response [122]. For driver alterations targeted by precision therapies, tracking individual VAFs can be expedient [123]; furthermore, a reduction in VAF has also been demonstrated to correlate with response to chemotherapy [124] and ICIs [[125], [126], [127]]. Liquid CGP assays have also investigated the aggregate signal across a sample's ctDNA content (*e.g.*, MSAF, or an absolute concentration of ctDNA) or other genomic features (*e.g.*, TF) to allow monitoring of less precise therapies such as ICIs [62], or in the setting of multiple potential resistance mechanisms. Sweeney *et al.* provided evidence that a reduction of ≥75% predicted response to enzalutamide in metastatic castrate-resistant prostate cancer [128].

In a landmark series of studies, a group studied serial ctDNA CGP in cohorts of patients receiving ICIs and found that a change in mean VAF correlated with benefits [125,129]. However, as discussed above, calculation based on mean VAF risks incorporating, along with tumor signal, other non-tumor variants such as germline mutation and clonal hematopoiesis of indeterminate potential (CHIP) [130]. In an updated analysis, the investigators showed that the correlation with outcome was improved using an analysis plan that was tumor-informed or avoided CH signals through PBMC sequencing [131]. Thus, the use of ctDNA CGP for monitoring must be done judiciously, favoring methods that can avoid CH signals which could confound interpretation of the results.

In advanced cancer monitoring, the ideal threshold for ctDNA reduction as a response indicator is a critical question. Research by the Friends of Cancer Research showed that a 50% reduction in maximum VAF was linked to positive outcomes in aNSCLC patients on immunotherapy [117]. However, for those receiving targeted therapy, complete ctDNA clearance was a better indicator [132].

Moreover, the timing of liquid biopsy sampling adds another layer of complexity to this puzzle. The BR.36 study found ctDNA levels fluctuating between detectable and undetectable at various points [133]. Similarly, the EMPOWER-Lung 1 study revealed that a 90​%+ ctDNA decrease signaled survival benefits at week 3 with cemiplimab, while complete clearance was important at week 9 [134]. Both studies suggest that patients who initially have ctDNA detected at an early on-treatment timepoint may have ctDNA not detectable at subsequent timepoints as their tumor responds.

It is evident that substantial ongoing research is imperative to validate the reliability of liquid CGP in monitoring cancers across various therapeutic modalities. Nevertheless, there is optimism that non-invasive disease monitoring through ctDNA analysis may complement traditional radiographic surveillance methods in a dynamic fashion, heralding a promising era in cancer care [135].

## 6 Technical differences between liquid CGP assays

### 6.1 Variable genomic coverage across CGP assays

Breadth of coverage is a basic way in which ctDNA CGP assays differ. Multiple panels emerged in parallel with the inclusion of key genes for a range of common cancer types, spanning up to 72 genes [[8], [9], [10]] or more [7,11]. Importantly, assays covering the same gene may have different degrees of genomic coverage across that gene; more comprehensive assays may include coverage of splice sites, introns, or local SNPs to enable improved detection of splicing alterations, rearrangements, and copy number changes. Sufficient representative genomic coverage offered by CGP is increasingly important with the emergence of genome-wide signatures such as tumor mutational burden (TMB) [[13], [14], [15]].

### 6.2 Variable bioinformatic approaches across CGP assays

CGP assays with similar genomic coverage can vary based on how the sequencing output undergoes bioinformatic analysis [136]. One clear area of variability is around germline alteration reporting [137]. Assays can also vary in their bioinformatic calculation of complex genomic biomarkers. Sturgill *et al*. analyzed real-world TMB results from 17,206 patients receiving liquid CGP: blood TMB (bTMB) was found to correlate well with tissue TMB and was most prognostic at a cutoff of 12 mut/Mb in one assay, whereas bTMB was discordant with tissue TMB and was most prognostic at 40 mut/Mb in the other assay [14]. Methods of genomic alignment and assembly can vary between assays as well. *De novo* assembly is computationally challenging but enables the discovery of novel sequences, such as rearrangements, that do not align to expectations defined by the panel.

## 7 Current challenges and future directions

### 7.1 Tissue CGP versus liquid CGP

**Fig. 2:**
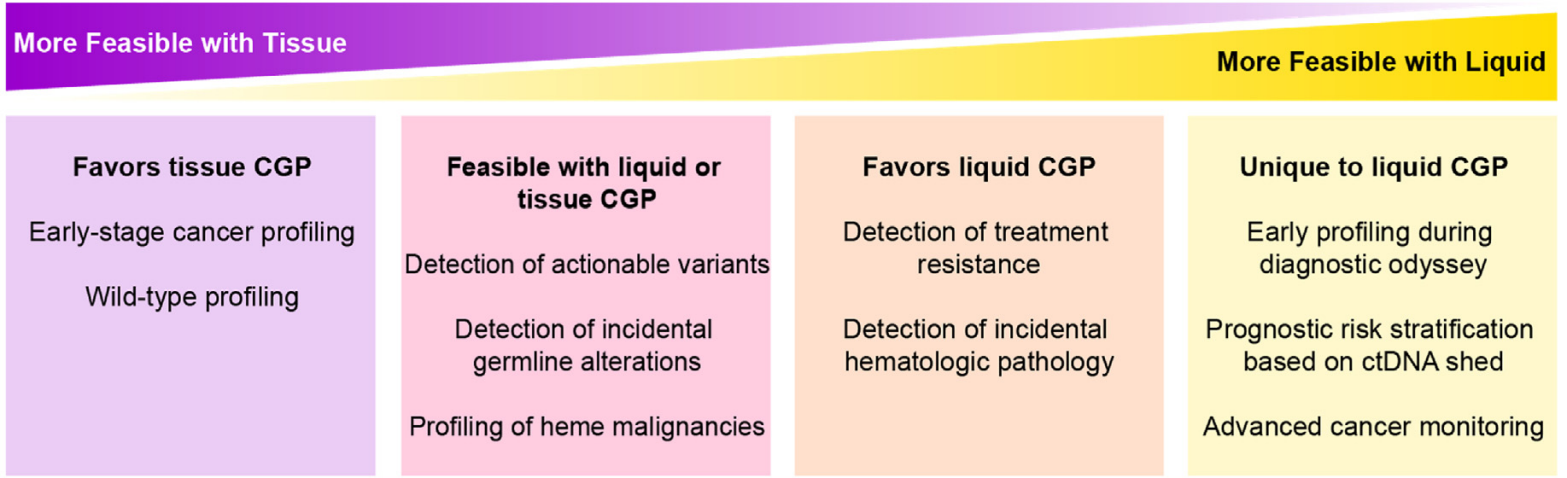
Relative feasibility of clinical use cases by sample type.

Tissue and liquid biopsy-based approaches are complementary, and both can be useful in clinical care. As reviewed elsewhere [19,138,139], there are multiple factors to consider when choosing between tissue and liquid biopsy, including tissue availability, urgency, and expected shedding of a tumor, as well as non-clinical factors, such as access and turnaround time. We categorized the use cases described above according to those favoring tissue or liquid, feasible with either, or unique to liquid CGP (Fig. 2).

### 7.2 Interpretation of a liquid CGP report

All of the use cases and findings described above impact how the results of a liquid biopsy-based CGP report must be considered. First, the presence of targetable drivers, especially those with FDA-approved companion diagnostic (CDx) claims, should be used to select therapy. The presence of resistance alterations should also be incorporated into therapy selection decisions. Clinicians must next consider the overall TF, as this offers insights into tumor burden, aggressiveness, and the negative predictive value (NPV) of negative results. Tumor burden and aggressiveness can inform risk stratification and therefore the need to escalate or de-escalate therapy regimens. The NPV of results increases when more ctDNA is present in the sample, particularly for short variants and rearrangements.

Further considerations involve the VAF of individual variants. A broad distribution of VAFs among different variants may be indicative of tumor heterogeneity or subclonality, as low-VAF variants may not be truncal drivers. A range of VAF values may also be due to CH, which is not targetable or representative of the tumor. A VAF of approximately 40%–60% may be consistent with a germline variant and therefore may have implications for testing by family members.

All of these considerations are further impacted by the size and composition of the panel of genes. As noted above, not all panels query all genes. While reporting a detected alteration clearly indicates that the gene was examined (even if not baited directly but instead found as a fusion partner via hybrid capture), the lack of a result may be because the gene was sequenced but no result was found, or because the gene was not sequenced. Here the multiple testing options available to physicians creates a challenge, as a lack of result on a given report requires confirmation of whether all genes of interest were in fact queried. The differences across disease types in which genes are relevant further complicates this problem. Confirmation of the panel make-up can typically be found as part of the report and/or from the vendor. Some reports helpfully note a short list of disease-relevant genes that were tested and/or where no alterations were detected.

Additionally, not all alterations are actionable. Indeed a large number of alterations are classified as Variants of Unknown Significance (VUSs) as defined by databases such as ClinVar [140]. These can add to the complexity reporting. All variants, including VUSs, should be reported, as future research may reveal a role in patient care. At the same time, larger gene panels will identify larger numbers of VUS alterations, which can potentially distract or confuse. Careful classifications of the potential pathogenicity of alterations, in addition to continuous upkeep of that database, must be combined with a clear and helpful report format to enable the best patient care while making all potentially relevant information available.

### 7.3 Future directions

Although the current landscape of liquid CGP predominantly revolves around blood-based testing, newer technologies aimed at assessing other bodily fluids are currently under development. This is particularly pertinent in cases where blood TF might be minimal (such as gliomas and central nervous system [CNS] disease) or where a more localized biological compartment proximal to the tumor site may enhance sensitivity and detection. Emerging evidence indicates the potential for alternative sources to enrich for fragments with genomic alterations from locoregionally present malignancies. This phenomenon has been observed in various bodily fluids, including stool (for colorectal cancer [141]), urine (for urothelial cancers [142]), cerebrospinal fluid (for CNS malignancies [143]), and pleural and bronchoalveolar lavage fluids (for lung cancer [144]), among others. To date, only ctDNA and circulating tumor cells (CTCs) are the components whose clinical application has been approved by the US FDA [145]. However, liquid biopsy analytes such as circulating tumor ribonucleic acids (ctRNAs), circulating tumor microRNAs, exosomes, extracellular vesicles, and metabolites may also be of interest. Research is underway on the use of ctRNAs as biomarkers, but the instability and extraction methods remain a barrier to scaling the method to allow for commercial CGP analysis [146]. To the best of our knowledge, no peer-reviewed evidence of feasibility has been reported yet.

Lastly, the increasing emphasis on artificial intelligence and machine learning may enable improved integration of liquid CGP in clinical treatment and decision making. Specifically, by utilizing computational algorithms and large-scale data analysis, machine learning may enable the development of predictive models for treatment response, which will further enhance the personalization of therapies for individual patients [147].

## 8 Conclusions

The unique opportunity of liquid biopsy is its potential to be deployed across multiple timepoints in the patient journey, meeting a wide array of diagnostic and treatment needs. Herein, with a focus on multigene liquid CGP-based assays, we detail one perspective illustrated by the “top ten” use cases that are emerging as indispensable tools in the clinical care of cancer patients. Yet, these use cases still likely represent only the tip of the iceberg, particularly considering the diverse ways in which ctDNA detection (rather than CGP) assays can be utilized, such as MRD and early cancer detection. As we work to increasingly adopt these flexible, patient-centered diagnostics into our clinical care and clinical research, we believe that the lexicon of use cases described here offers an opportunity for the liquid biopsy community to work toward a shared vision of future applications.

## Funding

Lincoln W. Pasquina, Candice Nulsen, Rachel B. Keller-Evans, Douglas A. Mata, and Hanna Tukachinsky are full time employees of Foundation Medicine, and this was part of their job to support. Aakash Desai and Geoffrey R. Oxnard were not paid specifically for this, and do not have funding for this.

## Author contributions

AD: Conceptualization; Writing - original draft; Writing - review & editing. LWP: Conceptualization; Funding acquisition; Project administration; Supervision; Visualization; Writing - original draft; Writing - review & editing. CN: Project administration; Writing - original draft; Writing - review & editing. RBKE: Visualization; Writing - original draft. DAM: Writing - original draft; Writing - review & editing. HT: Writing - original draft. GRO: Conceptualization; Funding acquisition; Resources; Supervision; Visualization; Writing - original draft; Writing - review & editing.

## Data sharing

Any unpublished data available confidentially upon request.

## Declaration of competing interest

AD serves on the advisory board for Amgen, Sanofi, Foundation Medicine, AstraZeneca, and Janssen Oncology. LWP, CN, RBKE, DAM, HT, and GRO were full-time employees of Foundation Medicine and owned stock in Roche Holdings AG at the time of writing the article.

## Abbreviations

ALK: anaplastic lymphoma kinase
aNSCLC: advanced non-small cell lung cancer
ASXL1: ASXL transcriptional regulator 1
ATM: Ataxia-telangiectasia mutated serine/threonine kinase
BRCA: breast cancer gene
BTK: Bruton tyrosine kinase
bTMB: blood TMB
CDx: companion diagnostic
cfDNA: cell-free DNA
CGP: comprehensive genomic profiling
CH: clonal hematopoiesis
CLL: chronic lymphocytic lymphoma
CNS: central nervous system
CSG: cancer susceptibility gene
CTC: circulating tumor cell
ctDNA: circulating tumor DNA
DNMT3A: DNA methyltransferase 3 alpha
ctRNA: circulating tumor ribonucleic acid
DNA: deoxyribonucleic acid
EGFR: epidermal growth factor receptor
ESR1: estrogen receptor 1
FDA: Food and Drug Administration
HRD: homologous recombination deficiency
ICI: immune checkpoint inhibitor
IDH2: isocitrate dehydrogenase 2
JAK2: Janus kinase 2
KRAS: Kirsten rat sarcoma
MDS: myelodysplastic syndrome
MRD: molecular residual disease
MUTYH: mutY DNA glycosylase
NGS: next-generation sequencing
NPV: negative predictive value
NSCLC: non-small cell lung cancer
PARP: poly (ADP-ribose) polymerase
PBMC: peripheral blood mononuclear cell
PFS: progression-free survival
PPM1D: Protein phosphatase, magnesium-dependent, 1, delta isoform
PPV: positive predictive value
RAF: rapidly accelerated fibrosarcoma
RAS: rat sarcoma
SF3B1: splicing factor 3b Subunit 1
SNP: single nucleotide polymorphism
TET2: Tet methylcytosine dioxygenase 2
TF: tumor fraction
TMB: tumor mutational burden
TMPRSS2-ERG: transmembrane protease serine 2:v-ets erythroblastosis virus E26 oncogene homolog
TP53: tumor protein P53
US: United States
VAF: variant allele frequency
